# Modulating Skin Aging Molecular Targets and Longevity Drivers Through a Novel Natural Product: Rose-Derived Polydeoxyribonucleotide (Rose PDRN)

**DOI:** 10.3390/cimb47120971

**Published:** 2025-11-23

**Authors:** Andrea Cavagnino, Gayané Azadiguian, Lionel Breton, Martin Baraibar, Annie F. Black

**Affiliations:** 1 OxiProteomics, 2 Rue Antoine Étex, 94000 Créteil, France; 2 Lancôme, 62 Quai Charles Pasqua, 92300 Levallois Perret, France; 3 CILIA Consulting, 20 Av De Paris, 78000 Versailles, France

**Keywords:** skin aging, skin photo-aging, natural product, PDRN, mitochondrial function, proteostasis, autophagy, skin longevity

## Abstract

Environmental stressors such as pollution and ultraviolet (UV) radiation contribute significantly to skin aging and skin photo-aging, alongside intrinsic chronological factors. Recent insights into longevity science have emphasized mitochondrial health, proteostasis, and autophagic balance as critical processes for maintaining skin integrity. This study investigates the protective potential of a natural product, Rose-derived PolyDeoxyRiboNucleotide (PDRN), against mitochondrial dysfunction and dysregulated autophagy in primary human keratinocytes subjected to environmental stress (benzo-a-pyrene and UV-A). PDRN was evaluated at 0.1%, 0.05%, and 0.01% concentrations. Mitochondrial function was assessed through membrane polarization, ATP/ADP ratio, Complex V (CV-ATP5A) levels, and citrate synthase levels. LAMP2A levels were quantified to evaluate the autophagic pathway. Complementary analyses were performed on ex vivo human skin explants, evaluating oxidative protein damage (carbonylation), Collagen I/III integrity, MMP1 and IL1a levels, and mitophagy markers (PINK1, PARK2). The results confirm significant protection of mitochondrial function, attenuation of oxidative stress, and modulation of autophagy-related pathways by PDRN across all models tested. These findings underscore the capacity of this novel natural product, a plant-derived PDRN, to mitigate environmental skin aging (and photo-aging) through mitochondrial maintenance and proteostasis regulation, positioning Rose-PDRN as a key active ingredient for dermocosmetic formulations targeting skin longevity biomarkers.

## 1. Introduction

Skin aging is a complex and multifactorial biological process driven by both intrinsic and extrinsic factors. Intrinsic aging is largely genetically determined and progresses with time, while extrinsic aging is primarily triggered by environmental stressors such as ultraviolet (UV) radiation, pollution, and oxidative stress. As the body’s primary protective barrier, the skin is in constant contact with these external aggressors, which accelerate aging processes by disrupting cellular homeostasis. The cumulative impact of intrinsic and extrinsic factors leads to progressive structural and functional deterioration of the skin, clinically manifesting as wrinkles, laxity, hyperpigmentation, and loss of barrier function, among others [1,2].

Among these extrinsic factors, fine particulate matter (PM10) and UltraViolet A (UVA) radiation are significant contributors to extrinsic skin aging and skin photo-aging. PM10, comprising particles with diameters of 10 μm or less, impairs the skin barrier, inducing oxidative stress, inflammation, and disruption of skin homeostasis. Concurrently, UVA radiation penetrates deep into the dermis, generating reactive oxygen species (ROS) that damage cellular components and degrade extracellular matrix proteins, leading to wrinkles, loss of elasticity, and pigmentation disorders [3,4,5].

The synergistic effect of PM10 and UVA exacerbates skin aging by amplifying oxidative stress and inflammatory responses. This combination not only accelerates the degradation of collagen and elastin fibers but also impairs the skin’s natural repair mechanisms, resulting in premature aging signs [6].

Beyond PM10, environmental pollution includes a class of polycyclic aromatic hydrocarbons (PAHs), among which benzo[*a*]pyrene (BaP) is a well-characterized example. These molecules are not only cytotoxic and mutagenic but also photosensitizing, meaning they absorb UV radiation and transfer the energy into biological tissues, producing heightened levels of ROS and cellular phototoxicity [7,8]. When present on the skin surface and exposed to UVA, PAHs undergo photochemical reactions that amplify oxidative stress, disrupt mitochondrial function, and further compromise cellular integrity [6,9,10].

The photosensitizing nature of PAHs renders them particularly dangerous in urban environments, where co-exposure to air pollutants and solar radiation is common. This dual insult causes mitochondrial and autophagic dysregulation, accelerating features of photoaging and potentially leading to chronic inflammatory skin conditions. Recent advances in the biology of aging have led to the identification of a framework known as the hallmarks of aging, which comprises a set of interconnected biological processes [11,12]. Among these, several are particularly relevant to skin: (a) mitochondrial dysfunction, which impairs energy production and increases reactive oxygen species (ROS), contributing to oxidative damage in skin cells, (b) disabled autophagy and mitophagy, which compromises the cell’s ability to clear damaged organelles and proteins, thus exacerbating cellular aging, (c) cellular senescence, characterized by stable cell cycle arrest and the acquisition of a senescence-associated secretory phenotype (SASP) that includes inflammatory cytokines (e.g., IL-1α), matrix-degrading enzymes (e.g., MMP1), that exacerbate local tissue inflammation, matrix degradation, and skin photo-aging phenotypes, (d) inflammation (*inflammaging*), as integrative hallmark, that further amplifies tissue degradation and accelerates senescence, (e) loss of proteostasis, including accumulation of oxidized proteins, that has also been identified as a key driver of aging phenotypes in the skin, (f) extracellular matrix (ECM) changes, characterized by a progressive reduction in ECM viscoelasticity, this hallmark directly accompanies the aging process in the skin, impacting tissue mechanical properties and cellular interactions [1,3,11,12]. Thus, maintaining cellular/tissular homeostasis in the face of these insults depends on tightly regulated processes involving energy metabolism, mitochondrial quality control, and autophagic flux, as well as on avoiding the formation of a damaged proteome, all of which are orchestrated by specific molecular markers.

Polydeoxyribonucleotide (PDRN) is a mixture of deoxyribonucleotide polymers typically known for its regenerative and anti-inflammatory properties [13]. It received increasing interest in recent years due to its therapeutic potential in tissue repair and dermatology [14,15,16]. Thanks to their strong wound-healing activity, PDRNs have been widely employed in skin regeneration, both as cosmeceutical agents and in medical esthetic applications. Since cutaneous aging shares common biological pathways with wound repair, the mechanisms activated by PDRN in tissue healing are also relevant for counteracting skin aging [17,18]. Furthermore, PDRN’s ability to modulate inflammatory responses and support DNA repair suggests it could be effective in mitigating environmental damage caused by pollutants and UV radiation, two major extrinsic aging factors [14,19,20]. Through these processes, PDRN can not only promote the regeneration of damaged cutaneous tissue but also help preserve skin integrity against external stressors. PDRN is usually obtained from salmon sperm, mainly *Oncorhynchus mykiss* and *Oncorhynchus keta*. However, this source is limited by seasonal availability, high cost, and ethical concerns. These limitations underscore the need for effective, sustainable, and ethically acceptable non-animal alternatives to traditional salmon-derived PDRN. Alternative origins have been explored, including marine organisms such as starfish (*Patiria pectinifera*) and sea cucumbers (*Apostichopus japonicus*), as well as botanical sources like red algae (*Porphyra* sp.) and *Panax ginseng*. These non-salmon PDRNs have shown antioxidant, anti-inflammatory, and regenerative effects comparable to those of fish-derived PDRN in skin models [17]. Thus, Rose-derived PDRN represents a novel, sustainable, plant-based alternative with promising dermocosmetic potential.

This study explores the protective role of rose-derived PDRN on mitochondrial function, autophagy, and oxidative balance in keratinocytes and human skin explants exposed to BaP/PM10 and UVA. By targeting hallmarks of aging, rose-derived PDRN emerges as a promising strategy to preserve skin longevity.

Mitochondrial function is central to skin health, supporting regeneration, differentiation, and extracellular matrix (ECM) maintenance. Key indicators such as mitochondrial membrane potential, ATP/ADP ratio, Complex V activity, and citrate synthase reflect cellular bioenergetics. Their decline is linked to accelerated aging, impaired turnover, and reduced barrier function [21,22,23]. Maintaining mitochondrial integrity is thus a major strategy to sustain skin longevity.

In parallel, autophagy, particularly mitophagy, ensures skin homeostasis by clearing damaged organelles and proteins. Chaperone-mediated autophagy (CMA), regulated by LAMP2A, protects cells from proteotoxic stress and contributes to mitochondrial quality control [24,25,26,27,28,29,30,31]. However, environmental stress and aging impair CMA, leading to protein aggregation, mitochondrial dysfunction, and reduced resilience.

Mitochondrial quality control also depends on PINK1/Parkin mitophagy and PGC-1α–driven mitochondrial biogenesis [32,33,34,35,36,37,38,39]. While these systems normally adapt to stress, prolonged exposure to UVA or pollutants can suppress them, compromising cellular defense and promoting premature senescence. PINK1/Parkin orchestrate ubiquitin-dependent mitophagy, targeting entire damaged mitochondria for macroautophagic clearance, whereas LAMP2A, the lysosomal receptor of CMA, mediates the selective degradation of soluble cytosolic proteins bearing KFERQ-like motifs. These pathways represent distinct arms of the autophagy network.

Environmental stressors such as UVA, PM10, and PAHs accelerate oxidative damage, upregulate MMP1, and trigger pro-inflammatory cytokines like IL-1α [40,41,42,43,44,45,46]. These processes fragment collagen fibers, weaken dermal structure, and amplify the senescence-associated secretory phenotype (SASP), key drivers of skin aging.

A further hallmark is loss of proteostasis, often measured by protein carbonylation, an irreversible oxidative modification [47,48,49,50,51,52,53]. Accumulation of carbonylated proteins reflects declining detoxification and clearance mechanisms, linking oxidative stress to matrix breakdown, inflammaging, and senescence.

This study demonstrates that plant-based PDRN extracted from *Rosa Hybrida* offers robust mitochondrial protection, supports autophagic homeostasis, and mitigates oxidative damage induced by environmental stressors. By addressing the fundamental biological processes, the root molecular causes of skin aging, rather than its superficial manifestations, these findings suggest an efficacious strategy for promoting long-term cutaneous resilience and longevity. Consequently, Rose-derived PDRN emerges as a new generation of longevity-inspired ingredients, designed to mitigate environmentally accelerated cutaneous aging while concurrently fostering regenerative capacity, delaying cellular senescence, and supporting long-term cutaneous health and longevity.

## 2. Materials and Methods

### 2.1. Rose-Derived PDRN, Material, DNA Extraction, and Characterization

Rose-derived PolyDeoxyRiboNucleotide (PDRN) is a complex mixture of deoxyribonucleotides isolated from the *Rosa* genus via an extraction process. Rose-derived polydeoxyribonucleotide (PDRN) was extracted from various parts of the *Rosa hybrida* (stems, leaves, flower heads) through conventional nucleic-acid extraction, purification, and fragmentation steps, following standard laboratory principles. The extracted Rose PDRN was characterized for its molecular properties and physical attributes [54].

### 2.2. Primary Cultured Keratinocytes

Keratinocytes (Biopredic International, Saint-Grégoire, France) were cultured in OxiProteomics^®^ medium at 37 °C in 5% CO_2_ humidified air. Cytotoxicity MTS assay (G542, Promega; Charbonnières-les-Bains, France) was carried out according to the manufacturer’s guidelines to determine the maximum tolerated concentration of PDRN at the appropriate time of contact with cells.

Rose PDRN was solubilized in medium (0.1%, 0.05%, 0.01% *w*/*v*) and applied for 48 h prior to stress exposure. For stress conditions, cells were exposed to Benzo[*a*]pyrene (BaP; CRM40071; 20 µM; Sigma-Aldrich-Merck KGaA, Darmstadt, Germany), diluted using Hank’s Balanced Salt Solution (HBSS), and irradiated with UV-A (LED source, emission peak at λ = 365 nm; 2.4 J/cm^2^) using the OxiProteomics^®^ irradiation system after contact with the rose extract. After irradiation, the medium was renewed, and the cells were incubated for 2 h before sampling. Controls received medium renewal only. Each condition was performed with N = 6 replicates (N = 3 across two independent experiments) for mitochondrial activity, LAMP2A, and ATP/ADP assays, and N = 3 replicates for other analyses.

### 2.3. Organotypic 3D Skin Model (Human Skin Explants)

Skin explants were obtained with the informed consent from abdominal surgery of a female Caucasian donor (44 years old, phototype II/III), distributed in 4 experimental groups (n = 3) and kept alive in a CO_2_-humid incubator. After initial equilibration of skin explants under optimal conditions, the rose PDRN was solubilized in (*w*/*v*) in ultra-pure water at defined doses (0.1% or 0.05%), topically applied on skin explants (30 µL/cm^2^), twice per day, for 3 days, before stress exposure. Then, after a gentle rinsing of the epidermal side with ultra-pure water, a solution (0.375 μg/cm^2^) containing particulate matter (Fine dust (PM10-like PAHs), ERM certified Reference Material; ERMCZ100, Sigma-Aldrich- Merck KGaA) was topically applied, and skin explants were exposed to UV-A (365 nm, 6 J/cm^2^). Just after irradiation, a fresh culturing medium was renewed. Topical application of ultra-pure water (vehicle) was performed for both the control and stress groups. The control group did not receive any additional treatment or stress exposure other than medium renewal. For sampling, half of each single explant was collected and included in OCT for cryopreservation. The other half was snap-frozen in liquid nitrogen and stored at −80 °C for molecular biology and biochemical studies.

### 2.4. Western Blot and ELISA Assays

Keratinocytes were subjected to protein extraction in an aqueous buffer using the optimized OxiProteomics^®^ buffer for Western blotting. Protein concentration was carried out using the Bradford Protein Assay Dye Reagent (Bio-Rad™, Marnes-la-Coquette, France) according to the manufacturer’s guidelines. Extracted proteins were separated by high-resolution electrophoresis (SDS-PAGE—gradient 4–20%; Thermo Scientific™, Thermo Fisher Scientific, Asnières-sur-Seine, France). After migration, proteins were transferred from the gel to a 0.2 μm nitrocellulose membrane (Bio-Rad™). The membrane was washed in Tris-buffered saline (TBS) solution at pH 7.6 with 0.1% Tween (TBS-T) before being incubated for 30 min in TBS with 3% Bovine Serum Albumin (TBS-BSA) for saturation. Just after the saturation step, the membrane was incubated at 4 °C overnight in a fresh TBS-BSA solution containing the primary antibodies (OXPHOS cocktail, Abcam, Cambridge, United Kingdom, ab110411-1001, targeting CV-ATP5A; Actin, Abcam, ab179467). The excess primary antibody was removed by washing with TBS-T solution, and then the membrane was incubated with the secondary antibodies coupled to a fluorophore (Anti-mouse Alexafluor 467, Invitrogen, Thermo Fisher Scientific, A21235 and Anti-Rabbit Alexafluor 488, Invitrogen, A11008). Additional washes with TBS-T were performed to remove the excess secondary antibodies. The digital acquisition of images of Complex V and Actin was performed using the “iBright” system (Thermo Fisher™, Thermo Fisher Scientific).

### 2.5. Mitochondrial Function Analysis via Mitotracker^®^

The mitochondrial function evaluation was conducted through an in situ treatment of cells with a Mitotracker^®^ probe, following the provider’s instructions (M22425, Red CMXRos; Invitrogen). The image collection of specific fluorescence signals was performed with the epi-fluorescence imaging system (EVOS M5000; Thermo Fisher). Image analysis was performed with ImageJ software (version 1.53, U.S. National Institutes of Health, Bethesda, MD, USA). The levels of fluorescent probe accumulation in active mitochondria were obtained by integrating the specific intensity of a signal normalized on the surface of the evaluation and N of the nuclei

### 2.6. ATP/ADP Detection and Quantification

Measurement of ATP (Abcam, ab83355) and ADP (Abcam, ab83539) levels was carried out using an ELISA kit accordingly to the manufacturer’s guidelines. The absorbance acquisition was performed using the “Varioskan” system (ThermoFisher).

### 2.7. Detection, Visualization, and Quantification of Biomarkers on Cells

Keratinocytes were fixed with an appropriate solution. Oxidatively damaged (carbonylated) proteins were labeled using a fluorescent probe functionalized to specifically bind to carbonyl moieties. For immunodetection of other biomarkers, a saturating step was carried out on the non-specific sites with a solution of PBS (Phosphate-Buffered Saline, pH 7.4; Sigma-Aldrich- Merck KGaA) containing BSA (Bovine Serum Albumin, Sigma-Aldrich- Merck KGaA). Cells were then incubated with diluted primary antibodies in a PBS-BSA solution (Table 1). DAPI (4′,6-diamidino-2-phenylindole) was employed for nuclear labeling. An appropriate secondary antibody conjugated with a fluorescent probe was then added (Table 1). The excess of probes or primary antibodies was eliminated with washing steps (PBS/BSA solution).

### 2.8. Detection, Visualization, and Quantification of Biomarkers on Skin Explant Sections

Explant sections of 5 µm in thickness were obtained using a cryostat (Leica, Wetzlar, Germany) and fixed with a solution. In situ carbonylation has been detected as previously reported [55,56,57]. For immunodetection, a saturating step of the non-specific sites was carried out with a solution of BSA in PBS. Tissue sections were incubated with a primary antibody for each independent biomarker detection in BSA/PBS solution (Table 1). The sections were incubated with the secondary antibody coupled to a fluorophore (Table 1). The cellular nuclei were labeled using DAPI (4′,6-diamidino-2-phenylindole). Washing steps for antibody excess removal employed PBS/BSA solutions. Fluorescent images were collected with an epi-fluorescent microscope (Thermo Fisher™, EVOS M5000 or M7000 Imaging System) and analyzed with ImageJ software.

### 2.9. Data Integration and Statistics

The quantification of biomarkers was normalized relative to the control (set at 100%), yielding a mean and standard deviation. Statistical analyses were carried out using the “GraphPad” software (version 10.0.3, La Jolla, CA, USA) by using a one-way ANOVA and Dunnett’s post hoc test for multi-comparison analyses (vs. the respective stress group) as well as Student’s binary *t*-test or Mann–Whitney comparisons between groups (* *p* < 0.05, ** *p* < 0.01, *** *p* < 0.001, ns not significant).

Two types of percentage-based metrics describing the relative effects of PDRN are reported:

(a) a baseline variation (% of induction) representing the stimulatory effect of PDRN, when applied without stress exposure, compared to the experimental group “control” was obtained by using the following equation:
  ***Baseline variation*** (*% of induction group* X vs. *group* Control) = ((*Biomarker_Levels_ group_*X/*Biomarker_Levels_ group_*Control) − 1) ∗ 100
(b) a protective value (% of efficacy) was used to describe the relative effect of PDRN under stress conditions and to indicate the positioning of each condition with respect to the non-stressed control (baseline control). This % of efficacy was calculated by setting the control group as the reference for maximum efficiency (100%) and the stress group as the reference for minimum efficiency (0%). Each experimental condition was then positioned within this range to provide a clearer interpretation of how PDRN counteracts stress-induced alterations relative to baseline. The following equation was employed for calculation:
  ***Efficacy*** (*% of efficacy group* X) = ((*Biomarker_Levels_Group_Stress* − *Biomarker_Levels_Group_* X)/(*Biomarker_Levels_Group_Stress* − *Biomarker_Levels_ group_*Control) ∗ 100

## 3. Results and Discussion

### 3.1. In Vitro Assessments (On Human Keratinocytes)

#### 3.1.1. Mitochondrial Membrane Polarization

**Figure 1:**
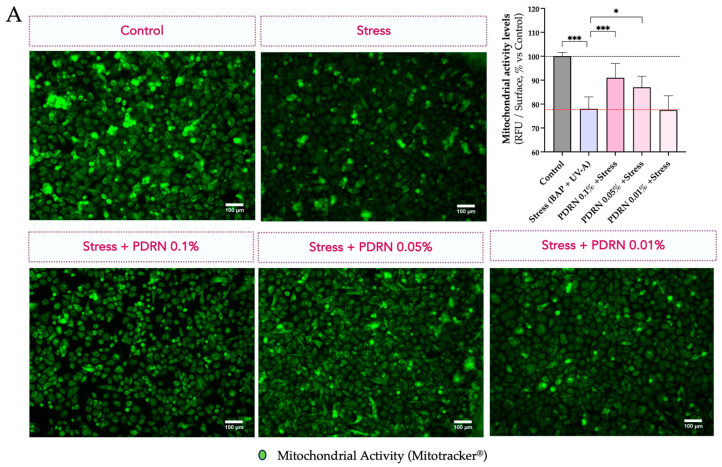

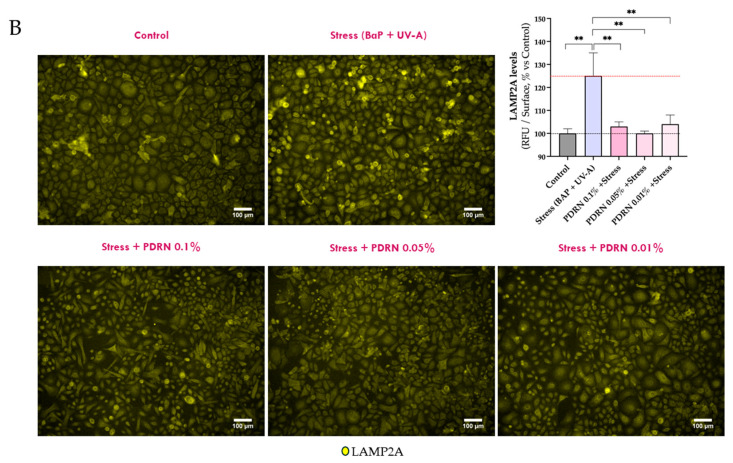
(**A**) The mitochondrial function is visualized in green by epifluorescence microscopy on keratinocytes and using Mitotracker^®^ probe that accumulates in active and functional mitochondria proportionally to their membrane polarization. The exposure to stress conditions (BaP and UVA) significantly decreased the fluorescence signal, while the presence of Rose PDRN preserved it and was associated with mitochondrial function. (**B**) LAMP2A levels are visualized in yellow by employing immunofluorescence labeling and epifluorescence microscopy. The stress induces an increase in LAMP2A levels, while Rose PDRN significantly contrasts this stress-induced modulation. Bar graph representations of mean values ± SD. Statistics: ANOVA and post hoc multi-comparisons versus Stress (* *p* < 0.05, ** *p* < 0.01, *** *p* < 0.001, not significant when not specified). Scale bar 100 µm.

As an initial exploratory evaluation, human primary keratinocytes were exposed to environmental stressors (BaP + UVA) and/or treated with Rose PDRN at three different concentrations in vitro. Human primary keratinocytes were exposed to BaP + UVA with or without Rose PDRN pretreatment. Mitochondrial function was assessed by Mitotracker^®^ fluorescence, reflecting membrane polarization. While Rose PDRN had no effect under basal conditions, the exposure to the stress conditions (BaP + UVA) induced a strong loss of mitochondrial polarization. Pretreatment with Rose PDRN preserved mitochondrial integrity, mitigating the negative effect of stress and indicating a beneficial effect on mitochondrial integrity and function under stress: 94% efficacy (0.1%), 100% (0.05%), and 88% (0.01%) (Figure 1A).

#### 3.1.2. Autophagy and Mitophagy

LAMP2A levels were monitored to assess autophagy, using in situ immunofluorescence (on cells) visualization and quantification (Figure 1B). BaP + UVA exposure significantly increased LAMP2A levels, consistent with the activation of autophagic mechanisms in response to cellular stress. Rose PDRN pretreatment prevented this increase, maintaining LAMP2A close to control values (89% at 0.1%, 100% at 0.05%, 86% at 0.01%), thereby supporting autophagic balance (homeostasis) and mitochondrial integrity.

#### 3.1.3. Mitochondrial Function and Content

**Figure 2:**
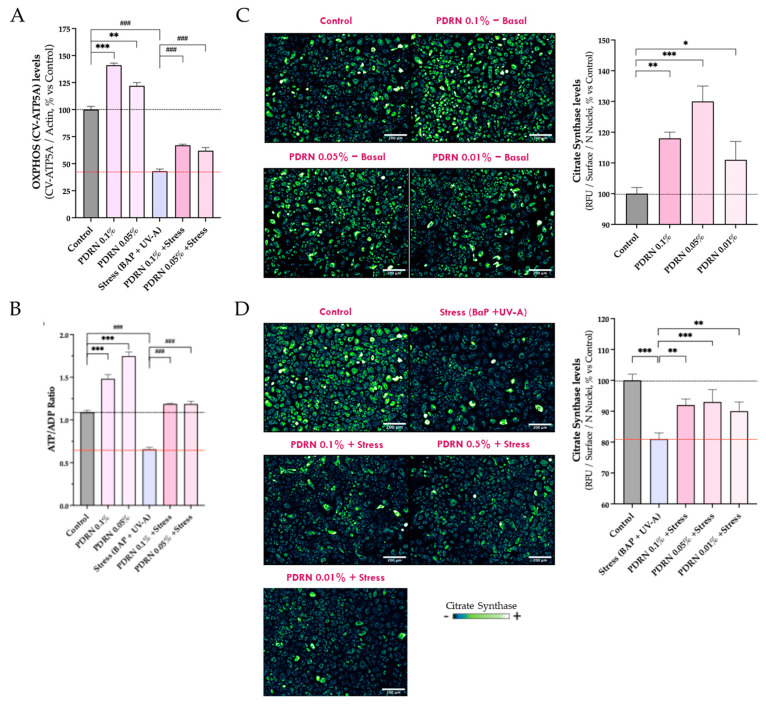
(**A**) The effect of Rose PDRN on oxidative phosphorylation is shown in histograms reporting mean values of Complex V levels (CV-ATP5A) assessed by Western blot analysis and normalization over Actin. (**B**) The effect on ATP/ADP (cellular energetic balance), assessed by ELISA, is shown by histograms. (**C**) The modulation of Citrate Synthase (CS) in basal conditions or upon stress (**D**) is visualized using a color scale in immunofluorescence images: darker colors indicate lower levels, while brighter colors represent higher levels. Visualization was performed using immunofluorescence labeling and epifluorescence microscopy. Rose PDRN stimulates basal CS levels and protects them from the negative effects of stress. Histograms show mean values ± SD. Statistics: One-way ANOVA followed by Dunnett’s post hoc multiple comparisons (vs. Control or Stress; * *p* < 0.05, ** *p* < 0.01, *** or ^###^ *p* < 0.001). Scale bar: 200 µm.

To further validate the beneficial effects of Rose PDRN on mitochondrial preservation, additional analyses were conducted using the 0.05% and 0.01% concentrations. Mitochondrial function was assessed by measuring CV-ATP5A, ATP/ADP ratio, and citrate synthase (CS) (Figure 2). Rose PDRN (0.05%, 0.01%) enhanced these parameters under basal conditions, increasing CV-ATP5A (induction of +41%, +22%), ATP/ADP ratio (+36%, +60%), and CS (+30%, +11%). BaP + UVA stress reduced all three markers, while Rose PDRN pretreatment significantly preserved them: CV-ATP5A (42%, 33% of protective efficacy), ATP/ADP ratio (total restoration towards the control), and CS (64%, 45% of efficacy). These results reinforce the ability of Rose PDRN to preserve mitochondrial bioenergetics and structural integrity under oxidative stress.

### 3.2. Ex Vivo (On Skin Explants) Assessments

#### 3.2.1. Mitochondrial Function

**Figure 3:**
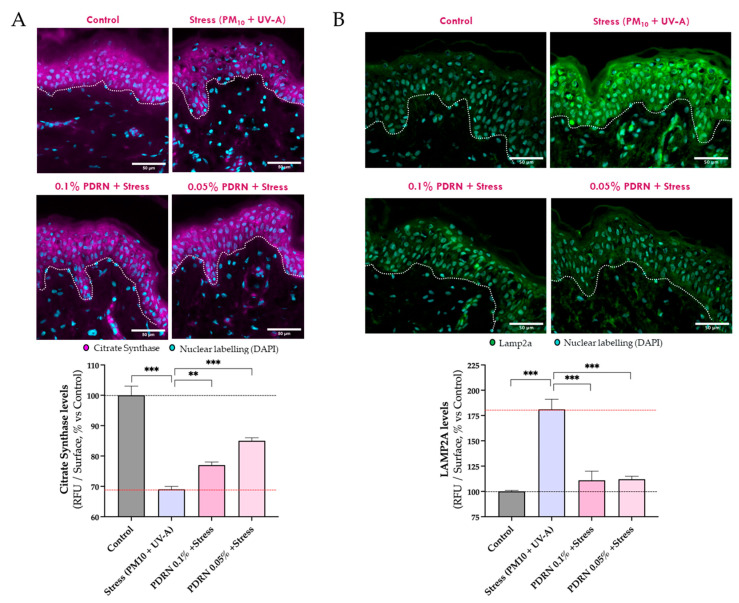
Citrate Synthase (CS) is visualized in magenta (**A**). LAMP2A is visualized in green (**B**). Immunofluorescence images show skin explants upon stress exposure. The nuclear detection (DAPI, in cyan) is superposed. Epidermis and dermis are separated by white dotted lines. The visualization was performed using immunofluorescence labeling and epifluorescence microscopy. Rose PDRN preserved CS levels from the negative impact of stress and contrasted the stress-induced increase in LAMP2A levels, which rises as a compensatory response to cellular stress. Statistics: ANOVA and Dunnett’s post hoc test versus Stress (** *p* < 0.01, *** *p* < 0.001, not significant when not specified). Scale bar 50 µm.

An ex vivo 3D human skin model was used to confirm Rose PDRN’s effects under PM10 + UVA stress. Citrate synthase (CS) levels, measured by immunofluorescence, were significantly reduced by stress but preserved with Rose PDRN pretreatment (25%of efficacy at 0.1%, 53% at 0.05%), consistent with the protective effects observed in keratinocytes (Figure 3A).

#### 3.2.2. Autophagy and Mitochondrial Homeostasis

In skin explants, the stress (PM10 + UVA) exposure significantly increased LAMP2A levels (Figure 3B). Rose PDRN pretreatment prevented this rise, preserving LAMP2A at 86% of efficacy of control values (for both 0.1% and 0.05%), indicating reduced need for compensatory autophagy and preservation of skin homeostasis and proteostasis.

Consistently, stress conditions in both keratinocytes and skin explants led to elevated LAMP2A levels, a marker of chaperone-mediated autophagy (CMA), suggesting a compensatory response to cellular damage. This damage was evidenced by increased oxidatively modified proteins (carbonylated species) under stress, as shown in Section 3.2.4. Under stress conditions, the pre-treatment with Rose PDRN normalized LAMP2A levels, likely reflecting a reduced requirement for CMA-mediated detoxification processes, in agreement with the decreased carbonylation observed in treated skin sections (detailed in Section 3.2.4).

**Figure 4:**
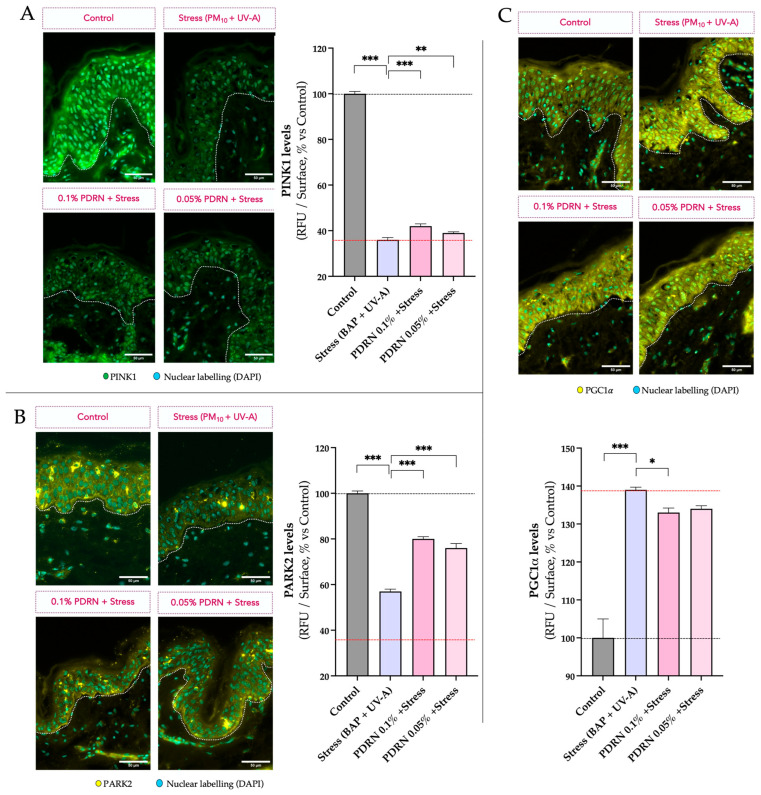
PINK1 signal on skin explant sections is visualized in green (**A**), PARK2 signal in yellow (**B**), and PGC1a signal in yellow (**C**). The nuclear detection (DAPI, in cyan) is superposed. Epidermis and dermis are separated by white dotted lines. The levels of each biomarker are presented in histograms as relative values and shown as mean ± SD for each experimental group. Rose PDRN significantly preserved the skin, mitigating the impact of stress exposure. Statistics: ANOVA and Dunnett’s post hoc test versus Stress (* *p* < 0.05, ** *p* < 0.01, *** *p* < 0.001, not significant when not specified). Scale bar 50 µm.

PINK1, PARK2, and PGC1α were subsequently evaluated via immunofluorescence analysis on skin sections (Figure 4A, B, and C, respectively) to further investigate the effects of the stressors and Rose PDRN on additional key markers of mitochondrial homeostasis. The stress (PM10 + UVA) reduced PINK1 and PARK2 levels, while Rose PDRN partially preserved them (PINK1: 9% of efficacy at 0.1%, 5% at 0.05%; PARK2: 54% and +45%, respectively) (Figure 4A,B). Mitochondrial quality control also depends on PINK1/PARK2-mediated mitophagy, which orchestrates ubiquitin-dependent clearance of damaged mitochondria through macroautophagy. Their significant decrease upon stress suggests impaired mitophagy capacity, while their partial preservation with Rose PDRN suggests improved mitochondrial surveillance and more efficient removal of dysfunctional mitochondria. This is consistent with the observed preservation of mitochondrial function and reduced stress-induced oxidative damage upon PDRN pre-treatment before stress exposure (following Section 3.2.4) in treated samples.

PGC1α levels were significantly increased by the stress exposure, while this increase was mitigated by Rose PDRN (16% at 0.1%), suggesting reduced mitochondrial damage and less need for compensatory biogenesis.

#### 3.2.3. Evaluation of SASP Markers and Dermal Matrix Integrity

To investigate the effects of Rose PDRN on SASP markers and dermal matrix integrity under environmental stress, the levels of IL-1α and MMP1, as well as the structural integrity of Collagen I and III, were evaluated in the ex vivo skin model by immunofluorescence analyses on skin sections.

**Figure 5:**
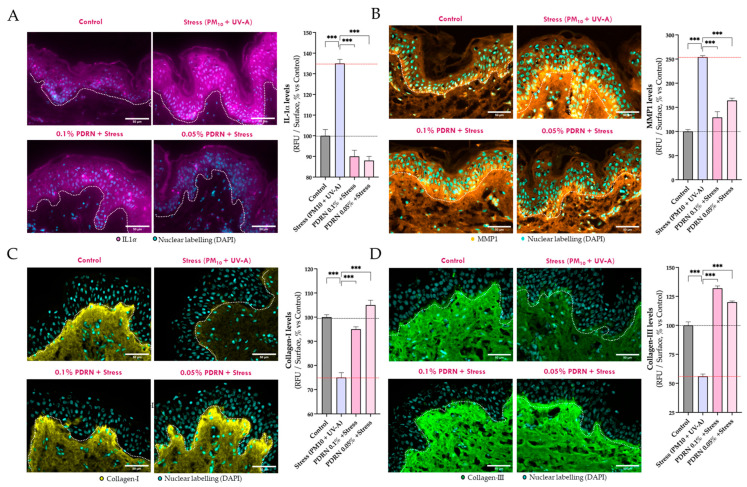
IL-1α signal on skin explant sections is visualized in magenta (**A**), MMP1 signal orange (**B**), Collagen I in yellow (**C**), and Collagen III in green (**D**). The nuclear detection (DAPI, in cyan) is superposed. Epidermis and dermis are separated by white dotted lines. The levels of each biomarker are presented in histograms as relative values and shown as mean ± SD, per each experimental group. Rose PDRN significantly counteracted the detrimental effects of stress exposure. Statistics: ANOVA and Dunnett’s post hoc test versus Stress (*** *p* < 0.001). Scale bar 50 µm.

As expected, IL-1α and MMP1 increased significantly upon stress exposure, while Collagen I and III were significantly reduced (Figure 5). Rose PDRN fully mitigated IL-1α induction (100% efficacy), significantly reduced MMP1 levels (81% at 0.1%, 58% at 0.05%), and preserved collagen integrity, maintaining Collagen I (81% at 0.1%, 100% at 0.05%) and Collagen III (100% at both concentrations). These findings highlight Rose PDRN’s ability to counteract SASP activation and confirm the potential of Rose PDRN to protect and maintain dermal structure.

#### 3.2.4. Proteostasis and Oxidative Stress

To further confirm the protective effects of Rose PDRN on cellular and tissue homeostasis, in situ protein carbonylation levels were assessed in skin sections.

**Figure 6:**
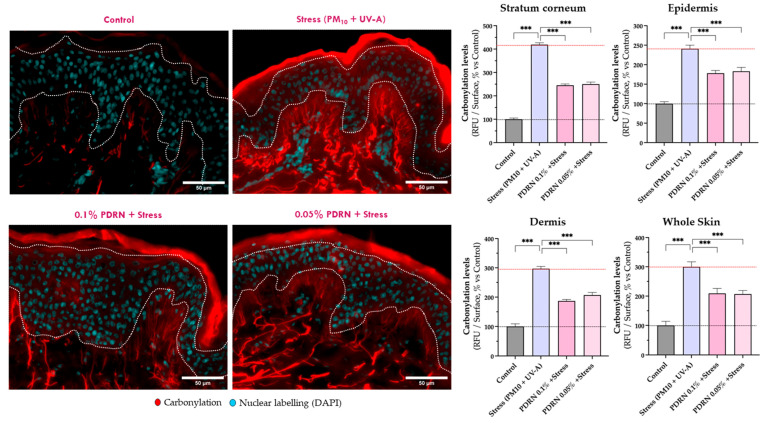
In situ visualization of Carbonylation levels (in red) by epifluorescence microscopy. The nuclear detection (DAPI, in cyan) is superposed. Epidermis and dermis are separated by white dotted lines. Stratum corneum, epidermis, and dermis are separated by white dotted lines. The quantified levels of Carbonylation of each experimental group are presented in histograms as relative values (% vs. Control) and shown as mean ± SD. The exposure to the stress (pollution; PM10 + UV-A) increased the in situ carbonylation levels in both the stratum corneum, epidermis, and dermis. Rose PDRN, at both tested concentrations, showed significant beneficial effects counteracting the impact of the stress (*** *p* < 0.001). Scale bar 50 µm.

As expected, exposure to stress resulted in a significant increase in protein carbonylation levels across all skin compartments (Stratum corneum, epidermis, and dermis) indicating a widespread oxidative damage that extends into the deeper layers of the skin. Rose PDRN pretreatment significantly reduced carbonylation in the stratum corneum (54%, 53% of efficacy), epidermis (45%, 41%), and dermis (56%, 46%) at 0.1% and 0.05%, respectively, resulting in an overall preservation of proteostasis of ~45–47% (Figure 6).

These results showed that Rose-derived PDRN effectively counteracts environmental stress–induced damage in both keratinocytes and human skin explants, highlighting the novelty of Rose-derived PDRN and confirming its interest as a plant-based alternative to conventional salmon-derived PDRN, thus offering sustainability and broad applicability in dermocosmetics.

## 4. Conclusions

This study provides insights into the cellular and molecular mechanisms through which a novel natural product, Rose-derived polydeoxyribonucleotide (Rose PDRN), exerts beneficial effects promoting skin longevity. Indeed, the results show that environmental stressors such as PM10, BaP, and UVA radiation, through the generation of reactive oxygen species (ROS), compromise mitochondrial function, autophagic balance, and proteostasis, which are recognized hallmarks of skin aging. Overall, Rose PDRN preserved mitochondrial polarization, energy balance (ATP/ADP), and key markers of mitochondrial function (CV-ATP5A, CS), confirming its role in maintaining cellular bioenergetics. Importantly, it also supported mitochondrial quality control by partially restoring PINK1/PARK2 expression and normalizing LAMP2A levels, suggesting balanced autophagy and mitophagy.

Beyond mitochondrial protection, Rose PDRN significantly reduced SASP-related markers (IL-1α, MMP1), preserved Collagen I and III, and decreased protein carbonylation across skin compartments, thereby maintaining ECM integrity and skin proteostasis. These effects collectively target multiple hallmarks of aging, including mitochondrial dysfunction, impaired proteostasis, inflammaging, and ECM degradation.

Together, these findings support this novel natural product (Rose-derived PDRN) as a promising longevity-inspired ingredient capable of protecting and preserving skin structure and function under environmental stress, offering potential for dermocosmetic applications aimed at long-term skin health and vitality.

## Figures and Tables

**Table 1:** List of antibodies.

Description	Target and Reference
Primary antibodies	Collagen I (Abcam/ab138492) Collagen III (Abcam, ab6310)
	IL-1alpha (Proteintech, 16765-1-AP)
	MMP1 (Abcam, ab137332) PINK1 (Abcam, ab216144) PARKIN/PARK2 (Abcam, ab77924) PGC1alpha (Abcam, ab191838) LAMP2A (Invitrogen, 51-2200) Citrate Synthase (Abcam, ab96600)
Secondary antibodies	Anti-mouse Alexafluor 647 (Invitrogen A21235)
	Anti-Rabbit Alexafluor 647 (Invitrogen A21244)

## Data Availability

The data are not publicly available due to confidentiality agreements.
